# Clinical outcome of laparoscopic versus robot-assisted radical cystectomy for patients with bladder cancer: a retrospective study

**DOI:** 10.1186/s12893-021-01382-1

**Published:** 2021-11-02

**Authors:** Yuchen Bai, Shuai Wang, Wei Zheng, EnHui Li, Jing Quan, Fei Wei, Qi Zhang, XiaoLong Qi, DaHong Zhang

**Affiliations:** 1 Department of Urology, Zhejiang Provincial People’s Hospital, People’s Hospital of Hangzhou Medical College, No. 138 ShangTang Road, Hangzhou, 310014 China; 2 Graduate Department, Bengbu Medical College, Bengbu, 233000 Anhui China

**Keywords:** Bladder cancer, Outcome, Laparoscopic radical cystectomy, Robot-assisted radical cystectomy

## Abstract

**Background:**

With the development of minimally invasive surgery technology, patients with bladder cancer are increasingly receiving laparoscopic radical cystectomy (LRC) or robotic-assisted radical cystectomy (RARC) treatment. The main purpose of this study was to compare the long-term outcomes of bladder cancer patients treated with LRC versus RARC.

**Methods:**

A retrospective study to identify patients with clinical stage Ta/T1/Tis to T3 bladder cancer who underwent RARC or LRC has been performed. The perioperative outcome, recurrence, and overall survival (OS) of the two surgical methods were compared.

**Results:**

218 patients were identified from March 2010 to December 2019 in our department, which including 82 (38%) patients who received LRC and 136 (62%) patients who received RARC. There was no significant difference between the two groups in terms of lymph node collection, lymph node positive rate, resection margin positive rate, and postoperative pathological staging. Compared with the LRC group, patients in the RARC group had a median estimated blood loss (180 vs. 250 ml; P = 0.02) and reduced complications at 90 days postoperatively (30.8% vs. 46.3%; P = 0.01). Recurrence, all-cause death, and cancer-specific death occurred in 77 (35%), 55 (25%), and 39 (18%) patients, respectively. The 5-year OS rate was 54.63% and 54.65% in the RARC and LRC group (P > 0.05). The 5-year cancer-specific survival (CSS) rate was 73.32% and 61.55% in RARC and LRC group (P > 0.05). There was no significant difference in OS [hazard ratio (HR) 1.083, 95% confidence interval (CI) 0.626–1.874; P = 0.78], and CSS (HR 0.789, 95%CI 0.411–1.515; P = 0.61) between two groups.

**Conclusions:**

Both RARC and LRC were safe and effective with a similar long-term clinical outcomes. Moreover, RARC had significantly lower median estimated blood loss and reduced postoperative complications.

## Background

Bladder cancer is the most common urinary system malignant tumor in China. According to the 2018 Global Cancer Statistics Report, bladder cancer has already become 8th in the rank of tumors. Approximately, 20–30% of patients have muscular invasive bladder cancer (MIBC). For patients with MIBC, radical cystectomy plus pelvic lymph node dissection is the standard of care. Open radical cystectomy is still the most common used approach of cystectomy in clinical practice. However, the complication of surgical procedure has a high perioperative complication rate and significant mortality [1–3]. In the past decades, the development of laparoscopic instruments and technology became mature, making laparoscopic as a new treatment option for patients with bladder cancer. At the same time, many advantages of the Da Vinci robot system has already showing up. Our department has carried out complete laparoscopic radical cystectomy plus orthotopic ileal neobladder since 2010, and robot-assisted radical cystectomy plus orthotopic ileal neobladder since 2014. Considering only few evidence on the comparison of the long-term clinical outcomes between patients treated with laparoscopic radical cystectomy (LRC) or robot-assisted radical cystectomy (RARC), this study aimed to investigate the perioperative and long-term outcomes of bladder cancer patients treated with LRC versus RARC.

## Materials and methods

### Patients’ screening

A retrospective study aimed to determine the clinical stage of Ta/T1/Tis to T3 bladder cancer patients receiving RARC or LRC. All patients received routine serum testing, abdominal or pelvic volume computed tomography (CT), and cardiopulmonary function testing. The individual patient’s disease characteristics and comorbidities, even the patient’s financial status, surgeon’s and patient’s preferences are recorded. This study was approved by the Ethics Committee of Zhejiang Provincial People's Hospital.

### Surgical process

All patients underwent general anesthesia. Operations were performed by three experienced surgeons for both laparoscopic and robotic-assisted operation. Both groups underwent radical cystectomy and systematic bilateral pelvic lymph node dissection with orthotopic ileal neobladder, and the urinary diversion was performed intracorporeally. The steps of surgical procedures were performed as previously reported [4, 5].

### Patients’ characteristics and outcomes

Patients’ characteristics including age, gender, body mass index (BMI), American Society of Anesthesiologists (ASA) score, previous medical history (smoking history, hypertension, diabetes, cardiac disease, and operation history), and preoperative neoadjuvant chemo-therapy were recorded. Perioperative outcomes including type of urinary diversion, operative time, estimated blood loss, rate of intraoperative trans-fusion and postoperative hospital stay were compared between two groups. The intra-operative and postoperative complications were classified according to the modified Clavien–Dindo classification system. Pathologic outcomes including total number of lymph nodes removed, surgical margin, and pathologic stage were also recorded and compared. The identification of cancerous cells at the level of the inked parenchymal excision surface was regarded as a positive surgical margin. All patients were tested with physical examinations, laboratory tests, chest radiography, and abdominal and pelvic ultrasonography or computed tomography during follow-up period. The follow-up arrangement was every 3 months for the initial 2 years, every 6 months for the next 2 years, and annually thereafter. Long-term outcomes including disease-free survival (DFS), overall survival (OS), and cancer-specific survival (CSS) were collected.

### Statistical analysis

Our research and data analysis follow the STROBE guidelines. Classification data is collected and analyzed as numbers and percentages. Standard deviation (SD) is used to check aggregated continuous data. Use the Chi-square test for categorical data and the Student’s *t* test or the Mann–Whitney U test for continuous variables to explore the difference between the LRC and RARC groups. OS is calculated from the day of surgery to the time of any cause-related death or the last follow-up. DFS is calculated from the day of surgery to the date of cancer recurrence or death from any cause. CSS is calculated from the date of diagnosis to the date of death due to bladder cancer. A Kaplan–Meier curve with a two-sided log-rank test and a Cox proportional hazard model with a calculated hazard ratio (HR) and 95% confidence interval (CI) were used to determine survival differences. All P values are two-sided and are considered significant when P < 0.05. All statistical analyses were performed using SPSS statistical software version 22.0 (SPSS Inc., Chicago, IL, USA).

## Results

### Patients’ population

218 patients were identified, including 82 (38%) received LRC and 136 (62%) received RARC. The median age and body mass index were 62 years old and 24.5 kg/m^2^. The majority of patients were male (76.1%), and the ASA score was low (1–2 points; 83.9%). Patient characteristics are summarized in Table 1. The distribution of age, sex, smoking history, BMI, American Society of Anesthesiologists (ASA) score, operation history, chronic disease was comparable between two groups. Only one patient (1.2%) in the LRC group and two patients (1.4%) in the RARC group had received neoadjuvant chemotherapy. The age, proportion of male patients, BMI, ASA score classification, past medical history, and neoadjuvant chemotherapy rate were also similar in the two groups.

**Table 1:** Patients characteristics

	Overall, n (%)	LRC, n (%)	RARC, n (%)	P
No. patients	218	82	136	
Median age, year (IQR)	62 (52–70)	65 (52–68)	63 (54–71)	0.54
Male patients	166 (76.1)	65 (78.8)	101 (74.2)	0.17
Median BMI, kg/m^2^ (IQR)	24.5 (22.3–27.1)	23.8 (22.1–26.8)	24.1 (22.6–26.9)	0.08
ASA Score				0.47
1	7 (3.1)	3 (3.6)	3 (2.2)	
2	176 (80.8)	66 (80.5)	108 (79.4)	
3	35(16.1)	13 (15.9)	25 (18.4)	
Medical history				
Smoker	80 (36.7)	29 (35.3)	51 (37.5)	0.22
Hypertension	61 (27.9)	26 (31.7)	35 (25.7)	0.54
Cardiac disease	26 (12.0)	12 (14.6)	14 (10.1)	0.16
Diabetes	28 (12.8)	10 (12.2)	18 (13.2)	0.43
Neoadjuvant chemotherapy	3 (1.3)	1 (1.2)	2 (1.4)	0.29
Surgical history	33(15.1)	17(20.7)	16(11.8)	0.33

### Perioperative outcomes

The operative time of RARC group was slightly longer than that of the LRC group, but the difference was not statistically significant. The median intraoperative estimated blood loss was 200 (range 100–480) mL and blood transfusion was performed in 24 (11%) patients. Patients received RARC had a significant less median estimated blood loss (250 vs. 180 mL; *P* = 0.02). The median hospitalization was 15 (13–22) days, and the intraoperative complication rate was presented in 11 (5%) patients. Intraoperative blood loss was higher in the LRC group than in the RARC group, but there was no difference in blood transfusion rate between two groups. The exhaust time was 2 (1–3) days, the feeding time of solid food was 4 (3–5) days, the total complication rate within 30 days was 28.0%, and the complication occurrence rate within 90 days was 37.2%. All patients were removed catheters and bilateral single J tubes after 13–15 days post-operation. There were no significant differences between the RARC and LRC group in terms of postoperative exhaust and feeding time, postoperative hospital stays, and the incidence of complications within 30 days after surgery. Of note, the incidence of complications and grade I–II complications within 90 days after surgery was dramatically lower in the RARC group than those in the LRC group (30.8% vs. 46.3%; P = 0.01), but there was no difference in grade III–IV complications (Table 2).

**Table 2:** Surgical outcomes and perioperative complications

	Overall, n (%)	LRC, n (%)	RARC, n (%)	P
Median operating time, min (IQR)	281 (229–400)	273 (225–410)	302(233–381)	0.28
Median estimated blood loss, mL (IQR)	200 (100–480)	250 (150–480)	180 (100–450)	0.02*
Intraoperative transfusion	24(11.0)	9 (11.0)	15 (11.0)	0.64
Median postoperative hospitalization, days (IQR)	15 (13–22)	15 (13–21)	16 (13–22)	0.59
Intraoperative complication	11 (5.0)	4 (4.9)	7 (5.1)	0.25
Exhaust time after surgery	2(1–3)	2(1–4)	2(1–3)	0.94
Food-taking time	4(3–5)	4(2–5)	4(3–5)	0.87
Post-operative 30-day complications	61(28.0)	28 (34.1)	33 (24.2)	0.15
Minor, Clavien–Dindo grade I–II	51(23.4)	22 (26.8)	29 (21.3)	0.19
Major, Clavien–Dindo grade III–IV	10(4.6)	6 (7.3)	4 (2.9)	0.24
post-operative 90-day complications	81(37.2)	38 (46.3)	43 (30.8)	0.01*
Minor, Clavien–Dindo grade I–II	64(29.4)	31 (37.8)	33 (23.5)	0.03*
Major, Clavien–Dindo grade III–IV	17(7.8)	7 (8.5)	10 (7.3)	0.08

*P value has statistically significant difference

### Pathologic outcomes

Pathologic outcomes for LRC and RARC were shown in Table 3. Positive nodes were identified in 9.6% and positive surgical margin was identified in 2.7% of all included patients. Lymph node yields with median node counts of 19 for LRC and 20 for RARC (P = 0.18). Postoperative tumor pathological stages revealed that 48 cases in Ta/T1/Tis stage, 134 cases in T2 stage, and 36 cases in T3 stage. There was no statistical difference in the number of lymph node dissection, positive lymph node rate, positive rate of resection margin and postoperative pathological stage between two groups.

**Table 3:** Pathological outcomes

	Overall	LRC	RARC	P
Median lymph node yield, (IQR)	19(14–24)	19(14–24)	20 (14–25)	0.18
Pathologic T Stage, n (%)				0.35
Ta/T1/Tis	48(22.0)	20 (24.3)	28 (20.6)	
T2	134(61.5)	49 (59.8)	85 (62.5)	
T3	36(16.5)	13 (15.9)	23 (16.9)	
Pathologic positive nodes, n (%)	21(9.6)	9 (9.8)	12 (8.8)	0.85
Positive surgical margin, n (%)	6 (2.7)	2 (2.4)	4 (2.9)	0.62

### Long-term outcomes

**Fig. 1:**
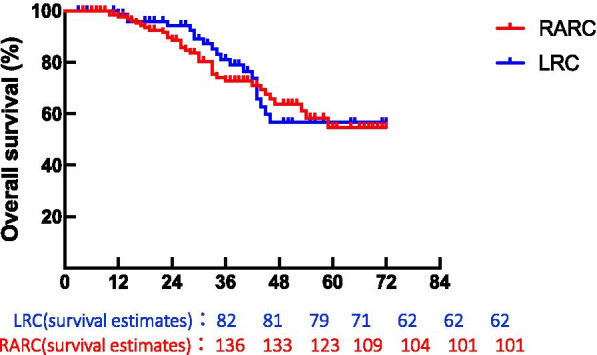
Kaplan–Meier curves of overall survival comparison in patients treated with RARC versus LRC. 5-years-OS = (RARC: 54.63%, LRC: 56.648%); P = 0.7757; HR = 1.083; 95CI = (0.6259 to 1.874)

**Fig. 2:**
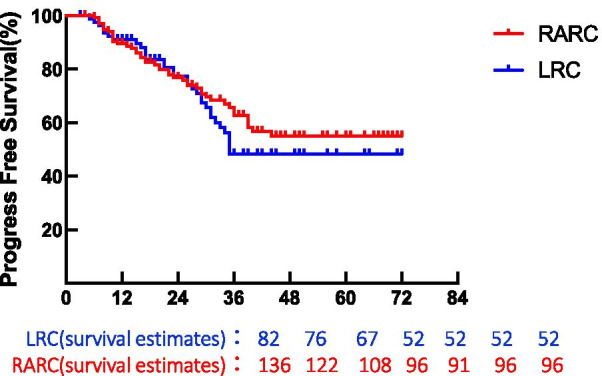
Kaplan–Meier curves of disease-free survival comparison in patients treated with RARC versus LRC. 5-years-PFS = (RARC: 55.066%, LRC: 48.175%); P = 0.6426; HR = 0.8297; 95CI = (0.5205 to 1.323)

**Fig. 3:**
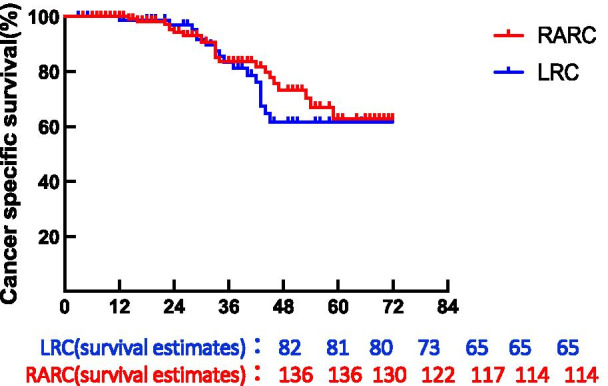
Kaplan–Meier curves of cancer-specific survival comparison in patients treated with RARC versus LRC. 5-years-CSS = (RARC: 73.318%, LRC: 61.554%); P = 0.6143; HR = 0.7891; 95CI = (0.4112 to 1.515)

The median follow-up was 33.0 months (IQR, 20.6–48.2 month). The recurrence, all-cause death, and cancer-specific death occurred in 77 (35%), 55 (25%), and 39 (18%) patients, respectively. The 5-year DFS was 55.07% in the RARC group and 48.18% in the LRC group. The 5-year OS rate was 54.63% in the RARC group and 54.65% in the LRC group (*P* > 0.05). The 5-year cancer-specific survival (CSS) rate was 73.32% in the RARC group and 61.55% in LRC group (*P* > 0.05). Importantly, there was no significant difference in OS (HR 1.083, 95%CI 0.626–1.874; P = 0.78) and CSS (HR 0.789, 95%CI 0.411–1.515; P = 0.61) between two groups (Figs. 1, 2, 3). There was 21 patients has been lost during the long-term outcomes.

## Discussion

Radical cystectomy was considered as standard-of-care for patients with MIBC [6]. Since 1990s, open radical cystectomy was the only surgical approach for the treatment of MIBC or high-risk non-MIBC. However, perioperative complication rate and mortality rate remained high and unsatisfactory due to the complicated surgical procedure. With the rapid development of minimally invasive techniques, LRC and RARC have become more and more important for the treatment of invasive bladder cancer [6] and have been extensively performed in many medical centers. The advantages of LRC and RARC included reduced complication rate and quick recovery post-operation [7–10], and the safety and efficacy of LRC and RARC are analogous to open radical cystectomy [11, 12]. Our department completed the first case of LRC and orthotopic neobladder reconstruction since 2010. Then, we gradually improved the details of the surgical procedure and began to perform RARC and orthotopic neobladder reconstruction since 2014. In clinical practice, we found patients received RARC had few blood loss and postoperative complications. To further compare the perioperative and long-term outcomes of these two surgical approaches in bladder cancer patients, we conducted this study to summarize the characteristics of the perioperative period and results of bladder cancer patients treated with LRC or RARC.

Although the RARC group reveals longer operation time comparted to LRC, it did not reach the statistical difference. Intraoperative blood loss was significantly higher in the LRC group then in the RARC group. Intraoperative blood transfusion rate and intraoperative complication rate were similar between two groups. In general, the difference between LRC and RARC might due to the inside and outside of the learning curve, surgeon’s experience, and the reasons for the renewal of surgical equipment. The difference in intraoperative blood loss seems related to different hemostatic methods and robotic instruments. The robotic surgery system has the characteristics of stable mechanical arm and lack of dead angle in the cavity. Whether it is separated or on the hemostatic clip, it could choose the best angle to cut into the desired level. In the narrow space condition, it would be better than laparoscopic surgery, and the high-definition three-dimensional vision system of the robot system could provide better anatomical structure and the direction of blood vessels to avoid damages [13–15]. Notably, the intraoperative, and postoperative complication rates were similar between two groups which were consistent with previous reports [16, 17]. There was also no difference on the intraoperative complication rate and the 30-day postoperative complication rate between two groups. However, patients in RARC group had markedly reduced 90-days postoperative complications than those in LRC group. Then we analyzed detailed data of 90-days post-operative complications. Most of the complications were urinary tract infection, pyelonephritis, lymphatic cysts, mild hydronephrosis, and small bladder stones. Most of them improved after conservative treatment or outpatient treatment. Additionally, we found that the potential reason for different complication rates between the two groups might be the improvement of the bladder suture methods. The RARC was mostly completed in the later period of the study. Several new methods including the U-shaped method could reduce the length of the intestine and maximize the length of the bladder, thus resulted in releasing the anastomotic tension between new bladder and urethra. The incidence of urinary fistula or obstruction would decrease. According to the previous experience, the new bladder is usually taken 30–35 cm ileum in the later period. The length-to-width ratio of the new bladder is about 4:3. During 3 months post-operation, the bladder volume will gradually expand after repeated expansion, then the bladder would be transforming to a new bladder with suitable volume and low pressure. Since the new bladder has relatively enough volume to keep the urine, it could reduce the urinary tract infection and protect renal function.

The reasons why patients received these operations had satisfactory outcomes and less complications may include: (i) the enlarged field of view under the endoscopic could provide satisfactorily view of neurovascular bundle and the urethra, which could reduce the damage of the ureter, keep better blood supply, and preserve appropriate ureteral length; (ii) the RARC could perform better quality anastomosed among urethra, ureter and new bladder. Complete operation inside the human body could minimize the anastomotic tension and prevent the twisting of the ureter or intestine; (iii) the single J tube inserted during the operation caused the new bladder under low pressure, which could prevent urinary leakage, reduce the probability of infection, ureteral stricture and reflux; (iv) we performed the new bladder with endovascular gastrointestinal anastomosis stapler, which could provide cutting and anastomosis been completed at same time; (v) the operation was completely performed in the abdominal cavity, avoiding the exposure of abdominal organs in the air which reducing the invisible water loss during the operation, the post-operation fasting time was shorter than before and the digestive function recovers quickly.

The postoperative pathology were mostly ≤ T2 stage (87.5%). The positive rates of lymph nodes were 9.8% and 8.8% in LRC and RARC, respectively. The positive rate of surgical margin was 2.4% and 2.9% in LRC and RARC, respectively. Compared with previous studies, the overall pathological results showed lower positive rates of lymph nodes and positive surgical margins [18, 19]. These differences might due to patients included in this study were all treated with orthotopic neobladder reconstruction. Thus mostly patients diagnosed with lower pathological stage before surgery. LRC and RARC found no difference in pathological T stage, positive lymph nodes or positive surgical margins, which is consistent with previous publication [20].

Although these results had several therapeutic implications, several limitations should be mentioned in this study. First, the sample size was small and the retrospective feature of this study will inevitably have selection bias. Thus, the results should be interpreted with caution and large-scale study especially a prospective one is still needed. Second, although the three surgeons are all experienced in endoscopic surgery, there were many other objective factors could influence the surgical outcomes [21–24]. RARC may get benefit from the late joined of the study which could affect the comparison results. Third, we did not record the functional data on daytime and nighttime continence. Last but not least, relatively shorter follow-up period may also become one of the limitations. Therefore, whether cystectomy under minimally invasive techniques and ileal orthotopic cystectomy can replace open surgery still remain undetermined [25–27]. Long-term, multicenter studies or randomized trials are still needed in the future.

## Conclusion

In summary, both LRC and RARC are safe and effective with a similar long-term clinical outcomes to treat muscular invasive or high-risk non-muscular invasive bladder cancers. And more, RARC has better performance than LRC in reducing intraoperative bleeding and postoperative complications. RARC had significantly lower median estimated blood loss and reduced postoperative complications. Whether RARC could replace LRC as a new standard surgical method for patients with bladder cancer need future investigation.

## Data Availability

The datasets analysed during the current study are available from the corresponding author on reasonable request.

## Abbreviations

LRC: Laparoscopic radical cystectomy
RARC: Robot-assisted radical cystectomy
OS: Overall survival
CSS: Cancer-specific survival
DFS: Disease-free survival
HR: Hazard ratio
95%CI: 95% Confidence interval
BMI: Body mass index
ASA: American Society of Anesthesiologists score
